# Nanosecond electron pulses in the analytical electron microscopy of a fast irreversible chemical reaction

**DOI:** 10.1038/s41467-019-11669-w

**Published:** 2019-08-13

**Authors:** Shyam K. Sinha, Amir Khammari, Matthieu Picher, Francois Roulland, Nathalie Viart, Thomas LaGrange, Florian Banhart

**Affiliations:** 1 Université de Strasbourg, CNRS, Institut de Physique et Chimie des Matériaux, UMR 7504, 67034 Strasbourg, France; 2 Laboratory for Ultrafast Microscopy and Electron Scattering (LUMES), École Polytechnique Fédérale de Lausanne (EPFL), 1015 Lausanne, Switzerland

**Keywords:** Structural properties, Imaging studies, Reaction kinetics and dynamics

## Abstract

We show how the kinetics of a fast and irreversible chemical reaction in a nanocrystalline material at high temperature can be studied using nanosecond electron pulses in an electron microscope. Infrared laser pulses first heat a nanocrystalline oxide layer on a carbon film, then single nanosecond electron pulses allow imaging, electron diffraction and electron energy-loss spectroscopy. This enables us to study the evolution of the morphology, crystallography, and elemental composition of the system with nanosecond resolution. Here, NiO nanocrystals are reduced to elemental nickel within 5 µs after the laser pulse. At high temperatures induced by laser heating, reduction results first in a liquid nickel phase that crystallizes on microsecond timescales. We show that the reaction kinetics in the reduction of nanocrystalline NiO differ from those in bulk materials. The observation of liquid nickel as a transition phase explains why the reaction is first order and occurs at high rates.

## Abstract (web summary)

Detailed knowledge of the transition states and kinetics of fast reactions in nanoparticles is desirable for many applications, but challenging to access. Here the authors obtain insight in nickel oxide reduction, using single-shot electron pulses in an electron microscope with nanosecond resolution.

## Introduction

The timescales of solid-state chemical reactions depend sensitively on the size and morphology of the reacting particles and on temperature. Reducing the size of solid reactants to nanometer dimensions leads to considerable shortening of the reaction times. Due to the increased importance of surfaces and interfacial area in nanoparticle systems, reaction kinetics may involve transition states that differ significantly from macroscopic solids, where reaction fronts proceed comparatively slowly due to diffusion-controlled mechanisms.

Detailed knowledge about the high-temperature kinetics and reaction speed in nanoparticles as well as transition states during the reaction is of high importance in various technical applications. Among the most important technologies are catalytic converters, where small metal particles on a substrate serve to transform gaseous environments at high temperature^1^. The particles are often exposed to undesired oxidation or to the deposition of solid reaction products that suppresses their catalytic function. The reduction of such oxide nanoparticles to pure metals is, therefore, an important process to re-establish their catalytic activity. Other applications of metal-oxide nanoparticles are also affected by oxidation, e.g., in solid oxide fuel cells^2^ or in the catalytic growth of graphene, carbon fibers and nanotubes^3^. Furthermore, the large magnetization of some transition metal nanoparticles is lost immediately upon oxidation, and the rapid reduction can be an indispensable reconditioning process for such applications. Understanding these transformations needs detailed knowledge about the reaction kinetics^4–6^ and the appearance of transition states during the reaction.

If the constituents are small, as it is the case in nanocrystalline materials, heating or cooling may be much faster than in macroscopic crystals so that high temperatures are reached immediately after a heat pulse. At temperatures around 1800 K, which is close to the melting point of many transition metals, the diffusion coefficients for lighter elements such as carbon vary within ~10^−9^–10^−15 ^m^2^ s^−1^. This leads to diffusion lengths of the same order as the size of nanocrystals (some nm to some tens of nm) within timescales ranging from the nano- to the millisecond. Concomitantly, diffusion-controlled solid-state reactions occur at much higher rates than in the bulk, and transition states only persist for nanoseconds to milliseconds. Hence, we need to study reaction kinetics in nanocrystals at high temperatures on their relevant, short timescales to determine salient mechanisms.

Despite the fundamental and technical importance of reaction kinetics in nanosystems, due to significant enhancement in reaction rates, experimental access remained difficult. The sub-millisecond analysis of reactions in nanometer-size specimens at high temperature has been an obstacle because the analytical tools require both high spatial with high temporal resolution. Optical spectroscopy with pulsed lasers or diffraction with X-ray pulses provide high temporal resolution but lack the necessary spatial resolution of electron microscopy techniques that can directly observe changes in the nanoparticles. Ultrafast (or dynamic) transmission electron microscopy (UTEM, DTEM) is a technique that enables us to achieve both high spatial and high temporal resolution in imaging and electron diffraction. The availability of electron energy-loss spectroscopy (EELS) also gives us access to the quantitative analysis of the elemental composition of the samples. High temporal resolution in imaging, diffraction, and EELS is achieved by using short electron pulses generated by laser photoemission. While reversible transitions can be studied in a stroboscopic experiment^7^, where a large number of pump-probe cycles are summed, the dynamics of irreversible reactions can only be observed with the single-shot approach^8–10^. It has been predicted that energy broadening effects in many-electron pulses can be severe^11^, making the application of EELS in the single-shot mode questionable. Since then, chemical information about the elemental distribution during fast irreversible transformations has yet to be demonstrated in dynamic TEM studies. This problem has recently been solved by using a modified photo-gun and TEM electron optical configuration to filter the electron pulses and improve their energy resolution while providing for high-collection efficiency in the spectrometer^12^.

Nickel oxide is an important model system for oxidation/reduction reactions. The existence of only one stable oxide and the single-stage transformation simplify the kinetics of the reaction. Nevertheless, the reduction kinetics of NiO is far from being understood, despite its industrial importance. The reduction of NiO by hydrogen^13–18^ or other gases^19,20^ has already been subject of several studies. However, there is a large variation in the reported activation energies, and literature about the carbothermal reduction is scarce^4^. Studies of the reduction kinetics of nanocrystalline NiO with high spatial resolution, e.g., by optical microscopy^21^ or in-situ electron microscopy^16^, have been carried out. Nevertheless, the determination of the exact mechanisms controlling the reaction kinetics are complicated due to the lack of high temporal resolution. This might be the reason why transition states haven’t been observed so far, despite their importance in the reduction kinetics. Furthermore, the use of conventional techniques requires us to arrest the reactions at defined, critical points and capture “snap-shots” of the phase concentrations, which is extremely challenging, if not, unfeasible. We need techniques that can directly observe these dynamics on their relevant timescales. Finally, there is almost no experimental knowledge about the kinetics of this reaction between the melting temperatures of Ni and NiO.

Here, we demonstrate that the time-resolved quantitative elemental analysis is feasible using the nanosecond, single-shot approach. We combine the EELS measurements with time-resolved electron diffraction and diffraction contrast imaging to study the fast irreversible reduction of NiO at high temperature induced by short laser pulses. By using 7 ns long electron pulses, we observe the fast reduction of NiO nanocrystals in contact with a carbon support layer and quantify the kinetics of the reduction process that occurs on a nanosecond to microsecond timescale. All measurements are taken during the ongoing fast reaction so that no intervention into the reaction process is necessary. Combining EELS measurements with diffraction and imaging techniques provides a detailed picture of the reduction reaction mechanisms in which the complementary data from each technique independently validates the timescales and quantification of the kinetics. Besides measuring reaction speeds, we show the presence of a short-lived liquid transition phase that decisively determines key pathways of the reaction kinetics.

## Results

### TEM with nanosecond electron pulses

Time-resolved TEM experiments are carried out in an ultrafast TEM^12,22^ where nanosecond electron pulses are used for imaging, electron diffraction, and EELS (see Methods Section). The experimental setup of the single-shot experiment has been described in detail previously^12^. A TEM with 200 kV acceleration voltage, equipped with an EEL spectrometer and a photoemitter (1 mm Ta disc), was used. In the present experiment, we used two nanosecond lasers with identical pulse duration of 7 ns for the pump-probe experiment. The lasers were synchronized by an adjustable electronic delay. The specimen was excited with 7 ns infrared pump laser pulses (1064 nm). Reproducible complete reduction of the NiO occurred at laser pulse energies of 90 µJ per pulse in the center of a Gaussian laser spot having a diameter of ~150 µm on the specimen. It has to be taken into account that only a very small fraction of the pulse energy is absorbed and heats the sample. Since an irreversible reaction is studied, the procedure had to be carried out in the single-shot approach^8,9,12^ where only one intense electron pulse is used. By setting adjustable delays between the sample laser heating pulse and the electron probe pulse generated on the photocathode by a 7 ns UV laser pulse (213 nm) from the second nanosecond laser, we studied the evolution of the reduction and transformation of the sample. Due to the unavoidable electron-electron interaction within the pulse, this entails several restrictions in spatial and energy resolution of the electron-optical system^12^ in comparison to standard TEM with continuous electron beams.

### Morphological evolution of the object

**Fig. 1:**
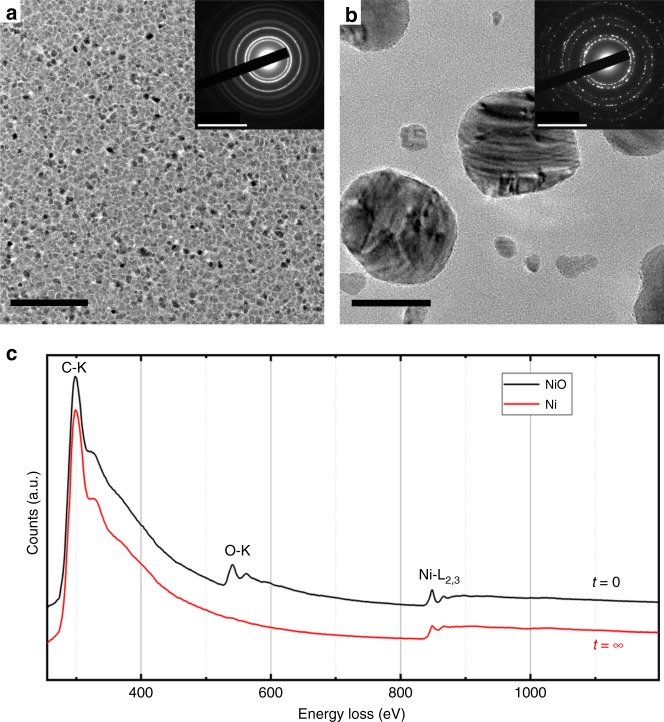
Morphology and composition before and after reduction. TEM analysis in imaging, electron diffraction **a**, **b**, and EELS **c** of the NiO crystallites before **a**, **c** and the Ni crystals after **b**, **c** reduction, taken with a continuous electron beam from plan-view specimens. Scale bars in **a**, **b**: 50 nm in the images and (10 nm)^−1^ in diffraction

The morphology of the polycrystalline NiO layer on the amorphous carbon membrane is shown in Fig. 1a in plan-view bright field imaging with a continuous electron beam. Crystallites of ~5 nm in size are visible. After exposing the system to a single IR pulse (1064 nm, 90 µJ) of 7 ns duration, pure Ni particles with a size distribution between 10 and 80 nm appear (Fig. 1b). The corresponding selected area electron diffraction patterns before and after the IR pulses with the different reflections of NiO (NaCl structure) and Ni (fcc) are shown in the insets. The NiOx nanocrystalline film fully transforms into isolated metallic nickel nanoparticles. The EEL spectra in Fig. 1c, obtained with the continuous electron beam, show the presence of the oxygen edge before and its absence after one IR pulse. It is therefore obvious that the reduction of the NiO layer occurs and is almost complete after one laser pulse.

### Temperature evolution during the experiment

The calibration of the specimen temperature after laser heating was undertaken by exposing the reduced Ni crystals (10–60 nm) on the C film (Fig. 1b) to IR pulses and determining the threshold for melting. This happened at laser pulses of 60 µJ, therefore the temperature after one 60 µJ pulse was close to the melting point of Ni (1728 K). For comparison, similar Ni particles on a Si_3_N_4_ film were heated with pulses up to 200 µJ but no melting occurred. Therefore, direct plasmonic heating of the Ni nanocrystals in the IR regime can be considered as low^23^, and we can assume that it is mainly the carbon film that absorbs the heat. Heating in the presence of the NiO layer is also due to IR absorption by the carbon film since the absorption of NiO in the IR is very low^24^. The reduction experiments were undertaken with 90 µJ pulses, but no signs of melting of NiO^25^ were found in diffraction patterns at all delays (see below). The specimen temperature after laser heating should therefore be between the melting temperatures of Ni (1728 K) and NiO (2257 K). Since the NiO layer vanishes at pulses above 90 µJ, an approximate specimen temperature of 2000–2100 K should be a realistic estimate.

**Fig. 2:**
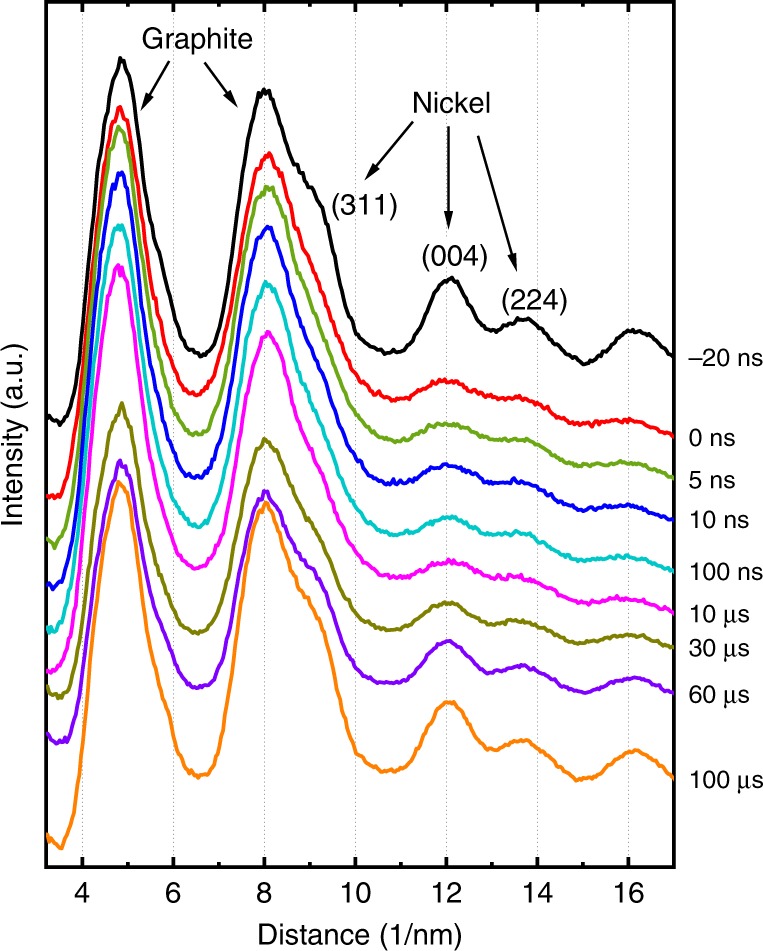
Melting and resolidification of Ni. Diffraction profiles taken from Ni particles on a carbon grid. The vanishing of the diffraction peaks from Ni shows the immediate melting of the particles within a few ns after the laser pulse. Each curve is the sum of 100 single-shot diffraction patterns at the same delay. The uppermost curve was taken 20 ns before the heating. The two prominent diffraction peaks at low diffraction angles are the graphite rings from the slightly graphitized carbon grid

The heating rate of the system under the laser pulses was measured by exposing Ni particles (after reduction of NiO) to IR laser pulses of different power and taking single-shot diffraction patterns at different delays. While pulses with a power below the melting threshold lead to thermal diffuse scattering around the diffraction spots, pulses above the threshold lead to melting of the Ni particles as observed from the disappearance of Bragg peaks and considerable broadening of the rings (Fig. 2). It is obvious that melting occurs faster than the time resolution of the setup (7 ns). This is seen as a weakening of the diffraction ring at zero delay (0 ns). Resolidification occurs after 60 µs which is in accordance with the results shown below. Thermal diffuse scattering (not shown here) shows the same temporal behavior; i.e., the immediate blurring of the diffraction spots.

### Time-resolved EELS, diffraction and imaging

**Fig. 3:**
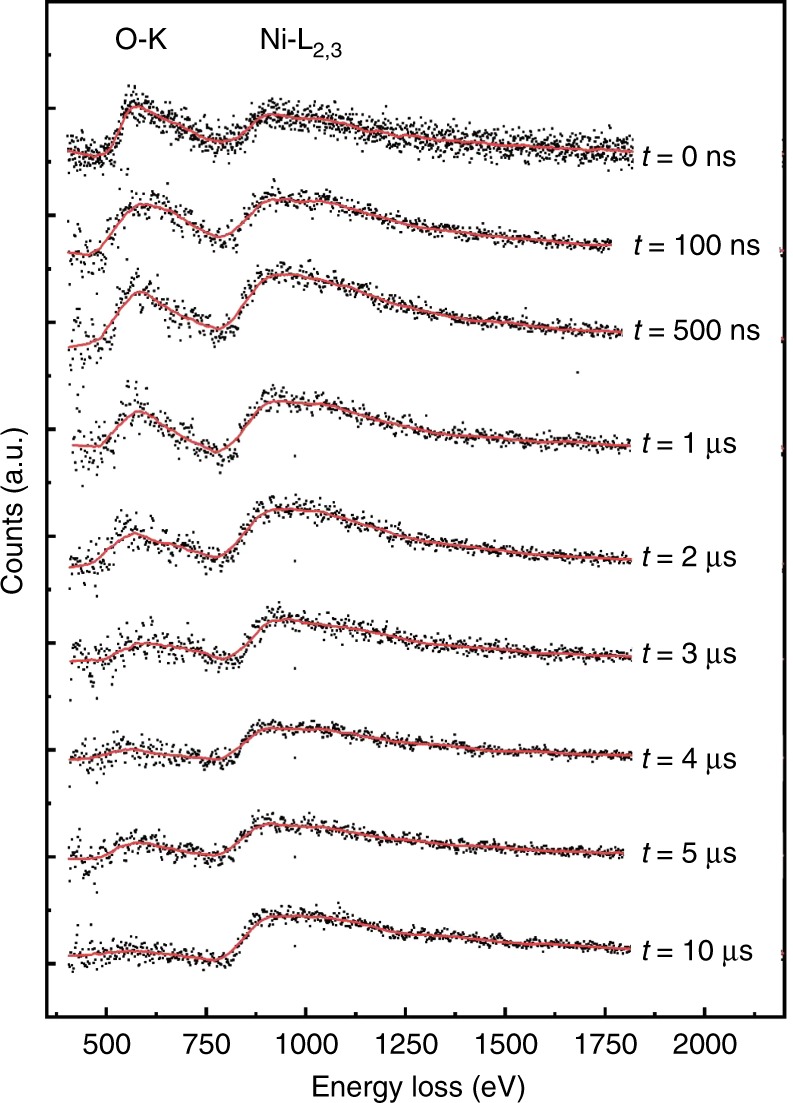
Time-resolved EELS. Electron energy-loss spectra taken with single 7 ns electron pulses at different times after the IR laser pulse

The evolution of the chemical reaction as a function of time was studied by taking EEL spectra in a series of experiments with a variable delay set between the IR pulse on the specimen and the electron pulse. The height of the oxygen edge relative to the Ni edge indicates the degree of reduction. Figure 3 shows a series of single-shot EEL spectra at delays between 100 ns and 10 µs together with the reference at *t* = 0 (pure NiO). The considerable decrease of the oxygen edge is observed between 1 µs and 4 µs.

**Fig. 4:**
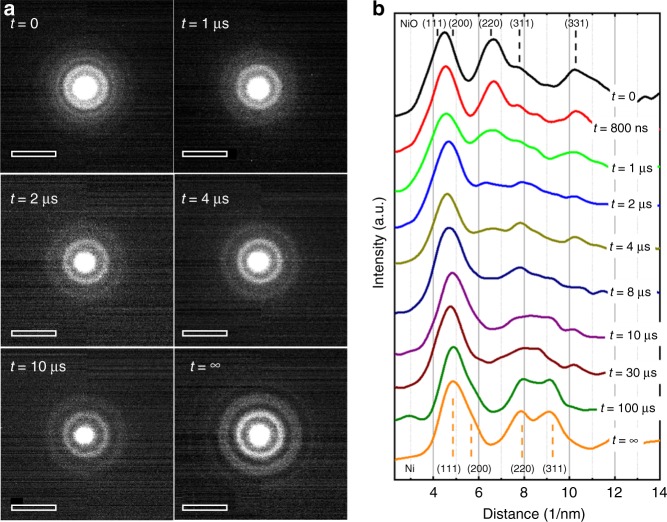
Time-resolved electron diffraction. Single-shot electron diffraction patterns taken from the layer system at different times after the laser excitation; **a** shows a panel of the diffraction patterns and **b** shows the radial profile through the patterns. The indexing of the peaks is given for NiO (top) and Ni (bottom). Scale bars in **a**: (10 nm)^−1^

The temporal evolution of the diffraction patterns is shown in Fig. 4a, in which each delay is a separate single-shot measurement taken on a fresh, unreacted area of the sample. Since the reacting system is deposited on an amorphous carbon film, the contribution from the underlying carbon support film to the diffraction pattern has to be removed. This is achieved by taking a single-shot diffraction pattern from the uncovered carbon support under the same conditions. Since the carbon film slightly transforms under the laser pulse (slight graphitization, seen in EELS and diffraction after less than 1 µs), the reference pattern has been taken after exposure to an IR pulse. The pattern from the carbon film was then subtracted from all further diffraction patterns. A montage of radial profiles through the diffraction rings, where an azimuthal integration around the circle has been taken^26^, is shown in Fig. 4b. Noise was removed by binomial smoothing filters. The (111)- and (200)-reflections from NiO (0.24 and 0.21 nm) are close to the (111)- and the (200)-reflections from Ni (0.20 and 0.18 nm), so the first broad diffraction peak around 5 nm^−1^ could not be reliably used for the analysis. The distinguishable reflections from NiO, the most prominent being the (220)- and (331)-reflections at 0.15 and 0.09 nm, show weak intensities at 1 µs and have almost disappeared at 4 µs. The first clearly distinguishable peaks of Ni correspond to the (220)- and (311)-reflections (0.13 and 0.11 nm) and are apparent at 10 µs but are still considerably broad. The profile of the Ni diffraction pattern peaks does not evolve after 100 µs, and the diffraction patterns show no more changes so that the transformation to solid Ni can be considered as complete.

**Fig. 5:**
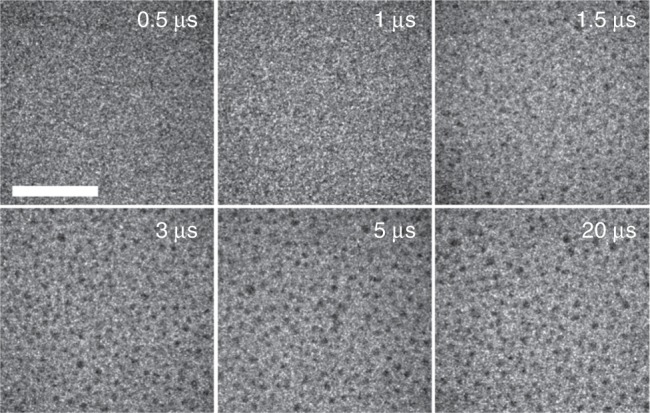
Time-resolved imaging. Single-shot TEM images in plan-view diffraction contrast at different delays (top right in each image). Only the Ni particles after the reduction are visible. Scale bar: 1 µm

Figure 5 shows a series of plan-view images in diffraction contrast, taken with single electron pulses after several delays. The limited resolution at low magnification and noise in the images does not permit imaging of the small 5 nm NiO crystals before reduction, and therefore no image contrast is visible for delays <1 µs. At 1.5 µs, contrast from the larger Ni particles starts appearing, but already after 3 µs no more significant changes in image contrast and the morphology of the nanoparticles are visible.

To compare the behavior of the system at short timescales with isothermal conditions close to equilibrium, the NiO-covered carbon films were heated in a standard heating stage at different temperatures in the range 570–630 K (see Supplementary Note 1). EEL spectra were taken with a continuous electron beam. The isothermal reduction occurs readily at a temperature of 580 K. At 630 K, the half-life of NiO was approximately 60 seconds. The results are shown in the Supplementary Figs. 1 and 2. The size and morphology of the Ni crystals after the slow reduction (Supplementary Fig. 1) are slightly different from the result after the laser-induced reaction (Fig. 1b). Although ripening has also happened, the Ni particles that remained solid during the process coalesced into a ramified structure. From the temperature dependence of the reaction speed, an activation energy of ~1.4 eV can be estimated for the reaction under isothermal conditions (see Supplementary Fig. 2).

To study the influence of the carbon film, similar experiments under laser pulses or continuous heating were also carried out with a NiO layer on a Si_3_N_4_ membrane where no carbon is involved (see Supplementary Note 1 and Supplementary Figs. 3 and 4). The irradiation with laser pulses did not lead to any detectable changes, which is expected since the IR absorption of both NiO and Si_3_N_4_ is very low. Under continuous heating in a heating stage, a slow and partial decomposition of NiO was observable. However, this happened at much higher temperatures than in a continuous heating experiment for NiO films deposited on carbon support films (Supplementary Fig. 2). The oxide crystals first coalesce, followed by a gradual reduction that becomes visible at 750 K. At 1070 K, the reduction is still incomplete after some minutes, showing that higher temperatures are needed for the direct reduction of NiO.

## Discussion

We first have to consider the timescales for heating and cooling of the system. The initial temperature of the system after the laser pulse is around 2000 – 2100 K. Heating in the laser pulse occurs from the carbon membrane which has a high IR absorption. Due to the initially very high temperature gradient between the carbon membrane and the NiO layer, heat conduction is very efficient, leading to a temperature rise of almost 2000 K in the NiO layer within approximately 1 ns, thus during the laser pulse (see the series of single-shot diffraction patterns in Fig. 2 and the calculation in the Supplementary Note 3). Heat transfer across the interface between the amorphous carbon film and the NiO crystals might be slower than between carbon and Ni but should also be much faster than the reduction kinetics. Since the total heat input to the system occurs before the onset of the reaction, the cooling rate is determined by the consumption of Gibbs free reaction energy during the reaction and radiative heat losses as well as lateral heat dissipation through the amorphous carbon film that also occurs after the reaction.

During the fast reaction, the system can be considered to a first approximation as adiabatic in which the Gibbs free reaction energy correlates to the heating or cooling of the layers. In bulk materials, the carbothermal reaction 2 NiO + C → 2 Ni + CO_2_ is endothermal with a reaction enthalpy of 87 kJ mol^−1^ and an activation energy of 3.3 eV^4^. However, in the present experiments, a nanocrystalline phase of NiO with a crystallite size of only 5 nm is used. It is known that the stability of nano-size materials differs considerably from bulk materials^27^. The high surface resp. interface energies change the reaction enthalpy as well as the activation energy. From the heating experiment under isothermal conditions (Supplementary Figs. 1 and 2), we measure an activation energy of 1.4 eV which is much smaller than the bulk value of 3.3 eV. This is understandable, given the large contribution of surfaces and interfaces in the nanocrystalline NiO layer. Extending the Arrhenius plot to higher temperature (Supplemetary Fig. 2c) and assuming a specimen temperature of 2000–2100 K, the reaction rate after a laser pulse is in agreement with the activation energy of 1.4 eV as obtained from isothermal annealing. The reaction enthalpy cannot be determined experimentally but should also be lower than the bulk value since the exothermal relaxation of the nanocrystalline NiO layer into the energetically lower bulk has to be considered. Considering the temperature before and after the reaction, the behavior of the system does not indicate a considerable contribution of the reaction energy. The starting temperature was not above the melting point of NiO that always remains in a crystalline state (Fig. 4), although, when first formed after the reduction, the Ni material is above its melting point. Therefore, a considerable contribution of the reaction energy to the temperature development within the reaction time (1–5 µs) has to be excluded.

The dominant mode of heat transfer after the laser pulse is radiative losses, whereas lateral heat conduction only has a minor influence (see details in the Supplementary Note 3). Cooling between the melting temperatures of NiO (2257 K) and Ni (1728 K) needs ~200 µs which corresponds well with the measured timescale for crystallization of the Ni particles of ~100 µs if we assume that the starting temperature was somewhat above the melting point of nickel.

**Fig. 6:**
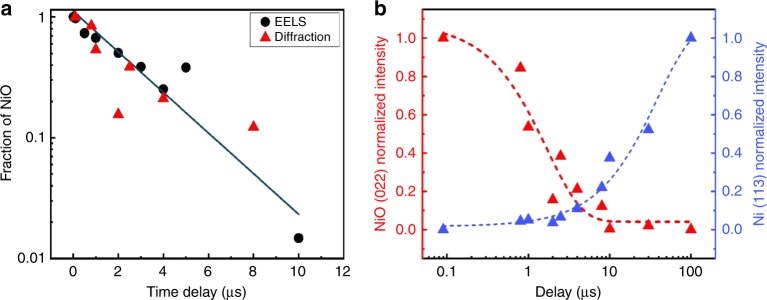
Decomposition of NiO. **a** Temporal evolution of the fraction of non-reduced NiO as taken from EELS (black dots) and from the intensity of the (022) reflection of NiO (red triangles). There is a larger uncertainty in the quantitative analysis at low percentages of NiO. **b** Evolution of the diffraction intensities of the (022)-reflection of NiO and the (113)-reflection of Ni

The fast reduction at high-temperature and its kinetics are studied by EELS, diffraction and imaging. However, only EELS data provide unambiguous information about the chemical composition during the reaction. Diffraction investigations show the appearance or disappearance of crystalline order and formation of transient phases while the imaging studies show the size and the distribution of larger particles on the carbon surface. It is an important observation that not all timescales measured with the three independent techniques are identical. The disappearance of the oxygen edge in EELS (Figs. 3 and 6a) corresponds well with timescale for the observed vanishing of the NiO reflections in diffraction (Figs. 4 and 6b) and the gradual appearance of the Ni particles in images (Fig. 5). This mostly occurs between 1 µs and 5 µs after the laser pulse. However, the diffraction peaks from crystalline Ni appear after the sample has sufficiently cooled (Figs. 4 and 6b), starting with a broadened peak at 8 µs until, at 100 µs, the diffraction peaks of Ni have reached their final shape. This indicates that the Ni particles are first in a non-crystalline state.

The temporary presence of a liquid Ni phase is probable since we determined that Ni particles already melt at laser pulses of 60 µJ, while a laser energy of 90 µJ effectively reduces the NiO layer in a single pulse. Therefore, the temperature when the reaction starts exceeds the melting temperature of the Ni particles that directly emerge from the reduction of the NiO crystals. The melting itself, which happens on the picosecond timescales^28^, would not be observable with nanosecond pulses but we can assume that the Ni immediately emerges in a liquid state from the reduction reaction. Since the Ni crystals are much larger than the NiO grains, a ripening process has occurred^29^. As seen in Fig. 5, the coalescence of the Ni particles happens in <3 µs which can occur with the diffusion rates of the liquid phase. We can therefore conclude that the reduction reaction is much faster than the successive coalescence and solidification of Ni so that the reduction reaction ends with Ni in a liquid phase. The successive cooling and solidification rates of the Ni crystals is determined by radiative heat losses of the layer.

Two reaction pathways have to be considered:

1. the direct decomposition 2 NiO → 2 Ni + O_2_ which is endothermic (240 kJ mol^−1^);
2. the carbon-assisted reduction 2 NiO + C → 2 Ni + CO_2_ which is less endothermic (87 kJ mol^−1^) with an activation energy of 3.3 eV^4^.

The question whether the reduction in this experiment is direct (1) or carbon-assisted (2) cannot be definitively answered by a simple EELS analysis. The overall loss of carbon during an experiment is small (see Supplementary Note 2) and partly due to thermal evaporation from the uncovered side of the carbon film. However, there are some qualitative arguments that favor the second pathway. The carbon-assisted reduction needs a lower reaction enthalpy and should therefore be favorable from an energetic point of view. The carbothermal reduction, being slower than the direct decomposition, starts at the hot carbon substrate and propagates by diffusion of carbon atoms through a liquid Ni layer and through grain boundaries of the nanocrystalline material. In the case of direct decomposition of NiO by the laser pulse, the reaction should start immediately in the whole layer and should be faster since it is not controlled by diffusion (heat transfer from the substrate to the surface occurs within a few nanoseconds). Furthermore, continuous heating at lower temperature shows that at the same temperature, the thermal reduction reaction in the presence of the carbon layer is much faster than on a Si_3_N_4_ layer. Therefore, the reaction barrier for the carbothermal reduction being lower is the most probable contributing mechanism in this study.

The EELS analysis (Fig. 6a) shows that the decrease of the oxide fraction after the IR pulse follows approximately an exponential law if we disregard statistical errors. This is typical for a reaction of first order. Assuming a first-order model for the decrease of the oxidized nickel fraction [NiO] = [NiO]_0_ exp(-*kt*), we obtain a reaction rate constant of approximately *k* = 3 × 10^5 ^s^−1^. A first-order reaction has already been reported for the carbothermal reduction of NiO^4^. The reduction should first be slower since the reaction at the solid-solid carbon-NiO interface is controlled by solid-state diffusion of carbon into the NiO. Once metallic Ni is formed, it is in the liquid state and covers the NiO grains. The reaction then proceeds by fast diffusion of carbon atoms through the liquid Ni layer. Since there is always an excess of carbon in the Ni solution (the carbon layer is only partially consumed in the reaction), we can assume that the carbon concentration remains almost constant. The reaction rate mainly depends on the NiO concentration that progressively decreases so that the reaction can be of first order.

Diffusion and solubility are the rate-limiting steps in the reaction which can be divided into the following partial reactions:

1. Diffusion of the first carbon atoms into NiO at the solid-solid interface and onset of the reaction.
2. Ni atoms aggregate and form a liquid Ni layer at the interface. CO_x_ vanishes by diffusion through the nanocrystalline NiO layer.
3. Carbon atoms diffuse through the liquid Ni layer until they reach the liquid-solid interface between Ni and NiO where the reaction proceeds.

It is apparent that the first two steps are less efficient since the diffusivity of carbon over the solid-solid interface is low. From the EELS data (Fig. 3), we see the onset of a considerable oxygen loss at ~1 µs after the heat pulse, which therefore corresponds to the duration of steps (1) and (2). Once a liquid Ni layer has formed, the reaction is getting efficient and speeds up due to the facilitated transport of carbon in liquid Ni^3^. During this period, the system follows a first-order reaction. Approximately 5 µs after the pulse, when the NiO is almost consumed, the reaction slows down. Such a sigmoidal behavior of the reaction rate as a function of time is in accordance with the Avrami model that has already been observed for the reduction of NiO^16^.

Taking all reaction steps together, we can conclude that the reactants have diffused over a length scale corresponding approximately to the thickness of the initial NiO layer (20 nm) resp. the liquid Ni layer at the interface (up to 10 nm). The averaged diffusivity *D* within the reaction time *t* = 5 µs over the distance *d* = 10 nm is therefore *D* = *d*^2^(2*t*)^−1^ *=* 1 × 10^−11 ^m^2^ s^−1^. This is less than expected for the diffusion of carbon in solid Ni at the melting temperature (2 × 10^−9 ^m^2^ s^−1^)^30^ or the diffusion of carbon in liquid Fe (data for liquid Ni aren’t available) just above the melting temperature (5 × 10^−9 ^m^2^ s^−1^)^31^. However, the solubility of carbon in Ni close to the melting point is approximately 10% which reduces the transfer of carbon through the Ni layer. Small-particle effects may also play a role in the reaction kinetics^32^. The most important observation is the temporary presence of the liquid transition phase of Ni that leads to an excess of carbon atoms during the reaction which then follows first-order kinetics.

It is now demonstrated that electron microscopy working with single nanosecond electron pulses gives detailed time-resolved information about the kinetics of fast reactions in nanomaterials. With the ongoing improvement of electron microscopy and EELS in the single-shot mode, a highly useful tool is available for detailed studies of the kinetics of fast reactions in nanomaterials. The combined quantitative analysis of the composition, crystallography, and morphology of the species during irreversible reactions is now possible at high specimen temperature where the reactions proceed accordingly fast. Detailed knowledge about the time dependence of reactions and phase transformations in nanocrystalline materials is of particular importance since reaction enthalpies as well as activation energies differ considerably from the bulk. By using the carbothermal reduction of nanocrystalline NiO as an example, it is shown that the reaction proceeds within 1–5 µs after a heat pulse and leaves liquid Ni as a transition phase that solidifies upon cooling of the system. With the observation of liquid nickel as a transition phase, the reaction of first order and, on longer time scales, of Avrami type can now be explained. Furthermore, we show that nanosecond laser pulses are a viable way of reactivating metallic nanoparticles after oxidation, and their use has the potential to be scaled to industrial processes.

## Methods

### Preparation of the nickel oxide films

Nickel oxide films were grown by Pulsed Laser Deposition technique (PLD) on amorphous carbon films of 20 nm thickness that served as substrates and were held by standard copper grids for TEM studies. With the direct deposition on a TEM grid, additional preparation steps could be avoided. A metallic nickel target is ablated using a KrF excimer laser (*λ*= 248 nm) with a 10 Hz repetition rate. The energy of the laser on the target was tuned to 1 J cm^−2^, and the distance between the target and the substrates was fixed to 5 cm. Nanocrystalline NiO layers of about 20 nm thickness with approximately 5 nm crystallite sizes (Fig. 1a) were then deposited under 0.1 mbar of oxygen at room temperature.

### EELS, diffraction and imaging

Quantitative information about the elemental composition and consequently the degree of oxidation was obtained by analyzing EEL spectra. Measuring the oxygen to nickel integrated intensity ratio as a function of time allowed us to follow the progressive decomposition of the oxide. Spectra were obtained by using an electron beam of 100 nm diameter (Supplementary Fig. 5) with an energy resolution in the spectrum of about 40 eV (this was sufficient for the quantitative analysis). The same experiment was repeated five times, and the spectra were summed to improve the signal-noise ratio. The transformation was also studied by time-resolved electron diffraction in such a way that patterns from small areas with many crystallites were taken (see Supplementary Fig. 6). By using a small condenser aperture and a parallel beam, the diameter of the electron beam spot was limited to 3 µm. The energy spread of the electron beam used to capture diffraction patterns was below 80 eV which was necessary to obtain acceptable statistics in the diffraction pattern but still allows identifying the different phases^12^. The spacings of the diffraction rings for NiO and Ni crystals could be distinguished, and traces for the temporary presence of transient phases were apparent. Complementary information was obtained by imaging with ns electron pulses. However, due to the unavoidable energy spread (65 eV in imaging), the influence of aberrations and image noise^12^, only moderate image resolution could be obtained. Hence, with EELS, electron diffraction, and imaging, three independent analysis techniques were combined to follow the temporal evolution of the reduction.

## Supplementary information

Supplementary Information

## Data Availability

The data that support the findings of this study are available from the corresponding author upon reasonable request.

## References

[1] Aiken JD, Finke RG (1999). A review of modern transition-metal nanoclusters: their synthesis, characterization, and applications in catalysis. J.Mol. Cat. A.

[2] Singhal SC, Kendall K (2003). High temperature solid oxide fuel cell - fundamentals, design and applications.

[3] Jourdain V, Bichara C (2014). Current understanding of the growth of carbon nanotubes in catalytic chemical vapour deposition. Carbon.

[4] Sharma SK, Vastola FJ, Walker PL (1997). Reduction of nickel oxide by carbon: III. kinetic studies of the interaction between nickel oxide and natural graphite. Carbon.

[5] Unutulmazsoy Y, Merkle R, Fischer D, Mannhart J, Maier J (2017). The oxidation kinetics of thin nickel films between 250 and 500 °C. Phys. Chem. Chem. Phys..

[6] Yu J (2018). Fast gas-solid reaction kinetics of nanoparticles unveiled by millisecond in-situ electron diffraction at ambient pressure. Angew. Chem. Int. Ed..

[7] Zewail A (2010). Four-dimensional electron microscopy. Science.

[8] King WE (2005). Ultrafast electron microscopy in materials science, biology, and chemistry. J. Appl. Phys..

[9] LaGrange T (2006). Single-shot dynamic transmission electron microscopy. Appl. Phys. Lett..

[10] LaGrange T, Campbell GH, Turchi PEA, King WE (2007). Rapid phase transformation kinetics on a nanoscale: Studies of the α → β transformation in pure, nanocrystalline Ti using the nanosecond dynamic transmission electron microscope. Acta Mater..

[11] Siwick BJ, Dwyer JR, Jordan RE, Miller RJD (2002). Ultrafast electron optics: Propagation dynamics of femtosecond electron packets. J. Appl. Phys..

[12] Picher M, Bücker K, LaGrange T, Banhart F (2018). Imaging and electron energy-loss spectroscopy using single nanosecond electron pulses. Ultramicroscopy.

[13] Richardson JT, Scates R, Twigg MV (2003). X-ray diffraction study of nickel oxide reduction by hydrogen. Appl. Catal. A.

[14] Janković B, Adnađević B, Mentus S (2008). The kinetic study of temperature-programmed reduction of nickel oxide in hydrogen atmosphere. Chem. Eng. Sci..

[15] Erri P, Varma A (2009). Diffusional effects in nickel oxide reduction kinetics. Ind. Eng. Chem. Res..

[16] Jeangros Q (2013). Reduction of nickel oxide particles by hydrogen studied in an environmental TEM. J. Mater. Sci..

[17] Zhou Z, Han L, Bollas GM (2014). Kinetics of NiO Reduction by H2 and Ni oxidation at conditions relevant to chemical-looping combustion and reforming. Int. J. Hydrog. Energy.

[18] Manukyan KV (2015). Nickel oxide reduction by hydrogen: Kinetics and structural transformations. J. Phys. Chem. C.

[19] Krasuk JH, Smith JM (1972). Kinetics of reduction of nickel oxide with CO. AiChE J..

[20] Alizadeh R, Jamshidi E, Ale-Ebrahim H (2007). Kinetic study of nickel oxide reduction by methane. Chem. Eng. Technol..

[21] Utigard TA, Wu M, Plascencia G, Marin T (2005). Reduction kinetics of goro nickel oxide using hydrogen. Chem. Eng. Sci..

[22] Bücker K (2016). Electron beam dynamics in an ultrafast transmission electron microscope with Wehnelt electrode. Ultramicroscopy.

[23] Link S, Burda B, Nikoobakht B, El-Sayed MA (2000). Laser-induced shape changes of colloidal gold nanorods using femtosecond and nanosecond laser pulses. J. Phys. Chem. B.

[24] Newman R, Chrenko RM (1959). Optical properties of nickel oxide. Phys. Rev..

[25] Yoo B-K, Kwon O-H, Liu H, Tang J, Zewail AH (2015). Observing in space and time the ephemeral nucleation of liquid-to-crystal phase transitions. Nat. Commun..

[26] Gammer C, Mangler C, Rentenberger C, Karnthaler HP (2010). Quantitative local profile analysis of nanomaterials by electron diffraction. Scr. Mater..

[27] van Steen E (2005). Stability of nanocrystals: thermodynamic analysis of oxidation and re-reduction of cobalt in water/hydrogen mixtures. J. Phys. Chem. B.

[28] Siwick BJ, Dwyer JR, Jordan RE, Dwayne Miller RJ (2003). An atomic-level view of melting using femtosecond electron diffraction. Science.

[29] McKeown JT (2012). Real-time observation of nanosecond liquid-phase assembly of nickel nanoparticles via pulsed-laser heating. Langmuir.

[30] Lander JJ, Kern HE, Beach AL (1952). Solubility and diffusion coefficient of carbon in nickel: reaction rates of nickel‐carbon alloys with barium oxide. J. Appl. Phys..

[31] Morgan DW, Kitchener JA (1954). Solutions in liquid iron. Part 3:—Diffusion of cobalt and carbon. Trans. Faraday Soc..

[32] Magnin Y, Zappelli A, Amara H, Ducastelle F, Bichara C (2015). Size dependent phase diagrams of Nickel-Carbon nanoparticles. Phys. Rev. Lett..

