# Supplementary material for: Nanosecond electron pulses in the analytical electron microscopy of a fast irreversible chemical reaction
Source: Nat Commun. 2019 Aug 13;10:3648. doi: 10.1038/s41467-019-11669-w (PMC6692388; doi:10.1038/s41467-019-11669-w)
Supplement: Supplementary file 1 — Supplementary Information [file 41467_2019_11669_MOESM1_ESM.pdf]

Supplementary Information

Nanosecond electron pulses in the analytical electron microscopy of a fast irreversible chemical reaction

Sinha et al.

Supplementary Information

Nanosecond electron pulses in the analytical electron microscopy of a fast irreversible chemical reaction

Shyam K. Sinha[1,a], Amir Khammari[1,a], Matthieu Picher[1], Francois Roulland[1], Nathalie Viart[1], Thomas LaGrange[2], Florian Banhart[1]*

[1] Université de Strasbourg, CNRS, Institut de Physique et Chimie des Matériaux, UMR 7504, 67034 Strasbourg, France

[2] Laboratory for Ultrafast Microscopy and Electron Scattering (LUMES), École Polytechnique Fédérale de Lausanne (EPFL), 1015 Lausanne, Switzerland

*florian.banhart@ipcms.unistra.fr

[a] These authors have equally contributed to the manuscript

Supplementary Notes

Supplementary Note 1: Isothermal reduction reaction at lower temperature

Heating of a NiO layer on amorphous carbon

To compare the activation energies of the fast reduction reaction after laser excitation with isothermal quasi-static conditions, the same system (i.e., a NiO layer on a carbon membrane) was heated in a standard TEM heating stage. Imaging, electron diffraction, and EELS were carried out with a continuous electron beam. From EEL spectra at different temperatures in the range 570 - 630 K, the quantitative composition of the system was determined as a function of time. Supplementary Figure 1 (see below) shows the NiO layer before and the Ni crystals after slow reduction at 630 K. Supplementary Figure 2 shows the temporal evolution of the composition as derived from a quantitative EELS analysis of the oxygen edge at different temperatures. An activation energy for the isothermal reaction of 1.4 eV is obtained. Extrapolating the line to 2000 K (Suppl. Fig. 2b), which corresponds to the reaction temperature after laser pulses, the measured reaction rate of the fast reduction (red dot) is in good agreement with the measurements under isothermal conditions (black dots).

Heading of a NiO layer on silicon nitride

The heating experiment was also carried out with a similar NiO layer of the same thickness (20 nm) and crystallite size on a **Si_3N_4** film. No carbothermal reaction is possible in this case. Supplementary Figure 3 shows TEM images and the according diffraction patterns before and after heating for several minutes at different temperatures. Supplementary Figure 4 shows an EELS before and after heating at 1070 K. Although a certain decomposition of NiO is visible, there is still a considerable percentage of oxygen up to 1070 K.

Supplementary Note 2: Loss of carbon in the IR laser pulses

To find out whether carbon loss due to ablation in the intense laser pulse has occurred, EEL spectra from a bare carbon membrane before and after a laser shot were taken. The loss of counts in the inelastic part of the spectrum relative to the zero-loss peak indicates a carbon loss of approximately 10%. Since the carbon membrane in the actual experiment is covered with the NiO layer on one side, half of the amount, i.e., a loss of 5% of the carbon layer by ablation can be assumed.

Supplementary Note 3: Heating and cooling of the specimen

Heating

Quantitative estimates:

The absorption of IR in NiO is very small, therefore heating of the NiO layer occurs by heat conduction from the hot carbon film during and after the laser pulse. The temperature change dT/dt at the surface of the NiO layer with thickness d is

$$\frac{dT}{dt} = \frac{\lambda}{C\rho}\frac{\Delta T}{d^2}, \quad (1)$$

where ΔT is the temperature difference between the carbon film and the surface of the NiO layer, λ the thermal conductivity, C the heat capacity, and ρ the density of the NiO layer. The temperature as a function of time is then

$$T(t) = T_{carbon}\left(1 - exp\left(-\frac{\lambda}{C\rho d^2}t\right)\right). \quad (2)$$

Assuming a temperature T_{carbon} = 2000 K of the carbon film, the specimen has heated by 1800 K within 1 ns.

Cooling

After heating, heat losses occur by the contribution of reaction enthalpy (see main text), heat radiation at high temperature, and lateral heat conduction through the layers.

Radiative heat losses follow dQ/dt = $\sigma\ \varepsilon\ A\ T^4$ and are the dominant mode of heat transfer at high temperatures (σ is the Stefan-Boltzmann constant, ε the emissivity and A the specimen surface). For the radiative cooling of the layer, we obtain a time span Δt between the initial and final temperatures T_1 and T_2:

$$\Delta t = \frac{2C\rho d}{3\sigma\varepsilon}\left(\frac{1}{{T_2}^3} - \frac{1}{{T_1}^3}\right). \quad (3)$$

Between the melting temperatures of NiO (2257 K) and Ni (1728 K) and assuming an emissivity of 0.8, cooling of the system by radiation would need approximately 200 µs. Assuming a slightly lower starting temperature, this is in accordance with the measured timescale for crystallization of the Ni particles of approximately 100 µs.

Heat losses by lateral heat conduction are less important due to the large heated area (150 µm in diameter) and the small thickness of the layers (altogether 50 nm). Due to the complicated geometry of the layer system on a Cu grid, a precise calculation is almost impossible; however, under some simplifying assumptions (no Cu grid, laser heats a cylindrical volume of the specimen), it is found that tens of milliseconds would be needed to cool the center of the specimen by 500 K. Therefore, the cooling after the reaction should be dominated by radiation.

Supplementary Figures:

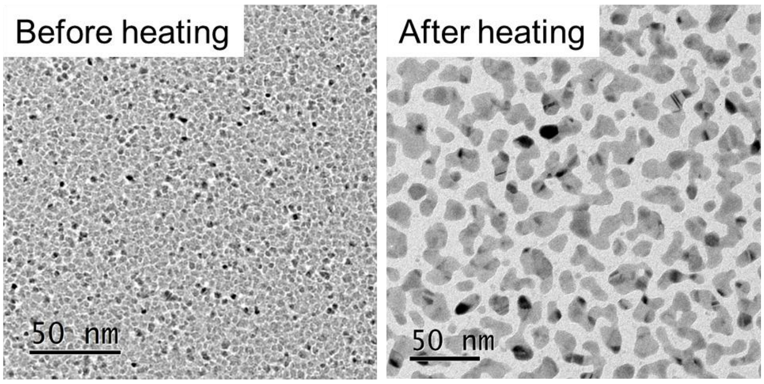

Supplementary Figure 1: NiO layer before heating and reduced Ni particles after heating at 630 K for some minutes.

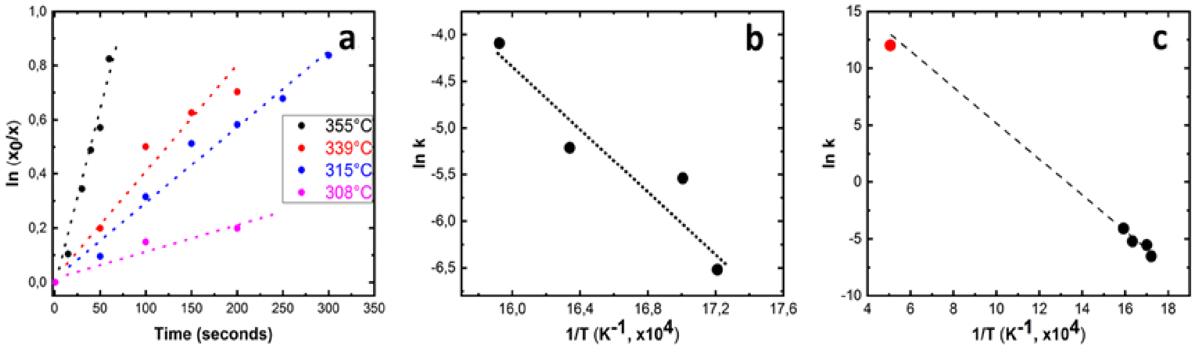

Supplementary Figure 2: (a) Concentration of NiO as a function of time during isothermal annealing at different temperatures. (b) The Arrhenius plot of the reaction rate as a function of temperature gives an activation energy of 1.4 eV. (c) Extrapolation of the line in (b) to higher temperatures. The red dot shows the measured reaction rate under pulsed heating and assuming a temperature of 2000 K.

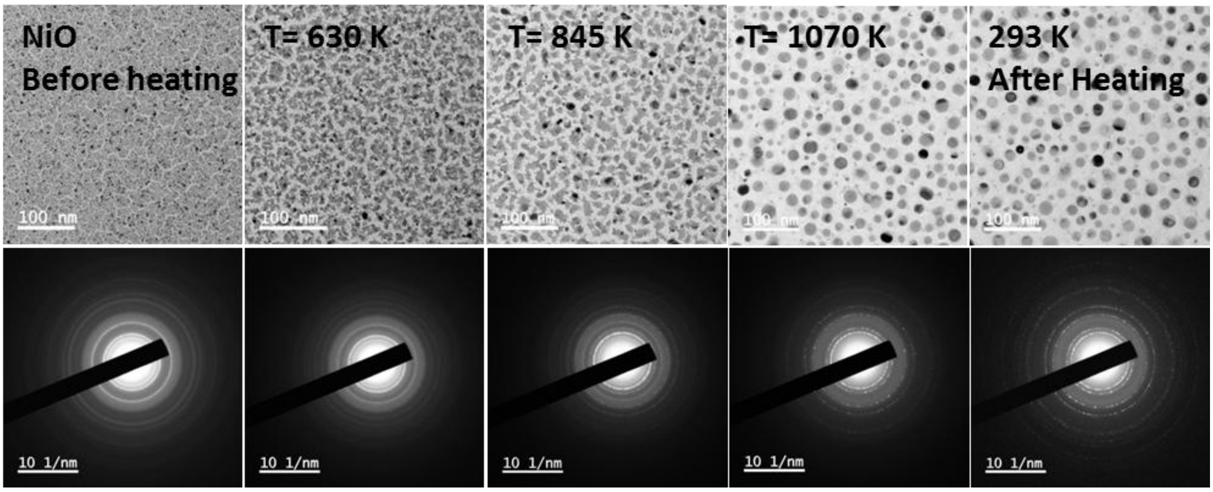

Supplementary Figure 3: Transformation of a NiO layer on a Si_3N_4 film under heating at different temperatures.

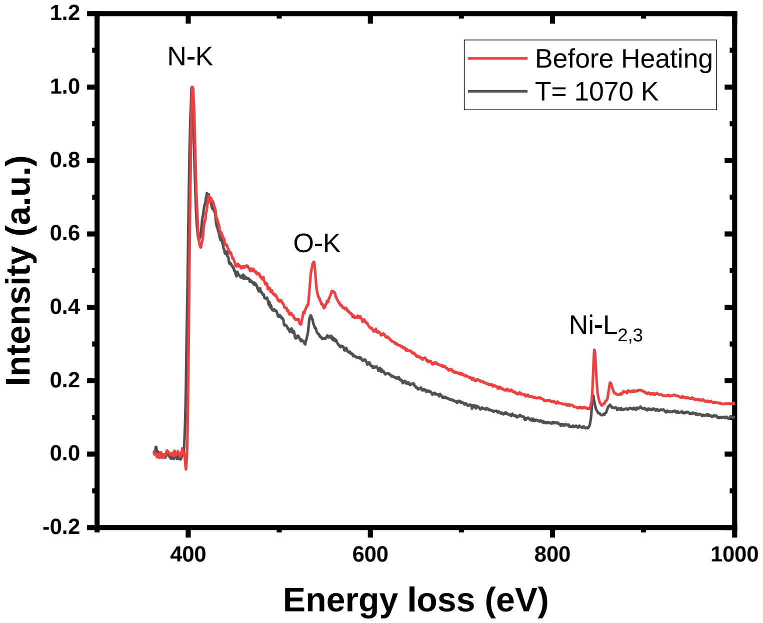

Supplementary Figure 4: EELS of a NiO layer on a Si_3N_4 film before and after heating at 1070 K.

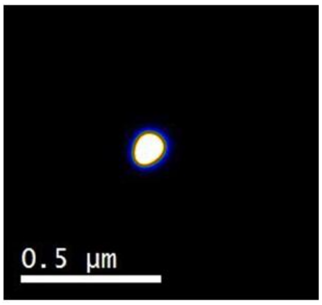

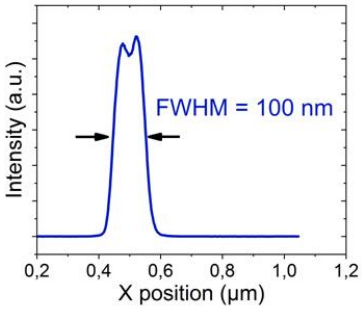

Supplementary Figure 5: Lateral electron beam profile of the single-shot electron pulses as used for EELS.

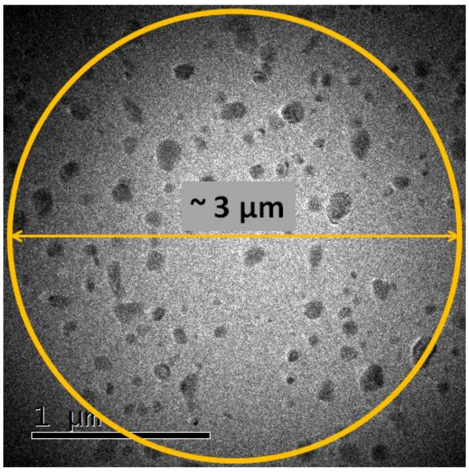

Supplementary Figure 6: Typical selected specimen area as used in single-shot electron diffraction.
